# Alcohol use disorders, beverage preferences and the influence of alcohol marketing: a preliminary study

**DOI:** 10.1186/s13011-020-00329-8

**Published:** 2020-11-30

**Authors:** Morgane Guillou Landreat, Céline Beauvais, Marie Grall Bronnec, Delphine Le Goff, Jean Yves Le reste, Delphine Lever, Antoine Dany, Karine Gallopel Morvan

**Affiliations:** 1 EA SPURBO 7479, Université de Bretagne Occidentale, Addictologie de liaison , Pole 3 , 1 étage, Hôpital de la Cavale Blanche , Bld Tanguy Prigent, 29200 Brest, France; 2 UMR 1246 SPHERE, University of Nantes / Tours, Tours, France; 3 HUGOPSY NETWORK, Rennes, France; 4 MD, Addictive disorders center, CH Morlaix, Morlaix, France; 5 CHU Nantes, Addictology and Psychiatry Department, Nantes, France; 6 MD, CHRU BREST, Addictology Unit CHRU BREST, Brest, France; 7 EHESP, School of Public Health, CREM UMR CNRS, 6211 Rennes, France

**Keywords:** Alcohol, Marketing, Advertising, Alcohol use disorders

## Abstract

**Background:**

Alcohol Use Disorders (AUD) are among the most prevalent mental disorders in the world. They are the leading risk factor for premature mortality and disability among 15 to 49-year-olds. Links between alcohol marketing and patterns of alcohol consumption are well defined in adolescents but there is few data on the impact of alcohol marketing on a population of drinkers with an AUD and seeking treatment. This study was designed in collaboration among researchers specialising in addictive disorders, in social marketing and primary care.

**Methods:**

This was a monocentric, cross-sectional, descriptive study. The main objective of this study was to define the type of marketing identified by drinkers with an AUD who were seeking treatment and their beverage preferences. Drinkers aged 18+ with an AUD and seeking treatment were included. A descriptive analysis and a logistic regression were carried out .

**Results:**

*N* = 91 patients were included, 73.6% were male, the average age was 46.2 years. 72% said they were not influenced by alcohol marketing, but 76% recalled an alcohol advertisement in the last 6 months. The most frequently reported beverage preferences were wine (39.6%), standard beers (29.6%), spirits (27.5%) and strong beers (16.5%).

**Conclusions:**

Patients with AUD, defined as vulnerable, reported exposure to alcohol marketing but did not seem to identify it consciously. Marketing influences differed according to beverage preferences. These results need to be confirmed by a larger study.

**Supplementary Information:**

The online version contains supplementary material available at 10.1186/s13011-020-00329-8.

## Introduction

Alcohol use is a leading risk factor contributing to the global burden of disease and causes substantial health damage [1–3]. 4.0% of the global burden of disease is attributable to alcohol [4]. Excessive consumption of alcohol induces 3.3 million deaths each year, 5.9% of all deaths [5].

Alcohol Use disorders (AUDs), defined by the DSM 5 [6], are characterised by impaired control over alcohol consumption and a chronic, escalating pattern of alcohol use despite significant damage affecting global health, the lives of family members and friends, and society in general [7]. AUDs are among the most prevalent mental disorders, particularly in high and upper middle-income countries [8, 9]. They are the leading risk factor for premature mortality and disability among 15 to 49-year-olds around the world [1]. In Europe, in 2010, the number of people affected by AUD was 23 million [10, 11]; it is one of the most important risk factors for morbidity, along with high blood pressure, tobacco and excess weight [12].

AUDs are complex, chronic disorders and the risk factors are individual, environmental and alcohol-related [7]. Factors associated with alcohol include alcohol availability (availability in shops, to minors, prices of alcoholic products), the position of alcohol in society and alcohol marketing strategies (i.e. advertising, sponsorship). From the public health perspective, these characteristics associated with alcohol are modifiable and are thus relevant for public health policies that combat alcohol misuse. For instance, the WHO (World Health Organisation) SAFER initiatives recommend **S**trengthening restrictions on alcohol availability, **A**dvancing and enforcing drinking counter-measures, **F**acilitating access to screening, brief interventions and treatment, **E**nforcing bans or comprehensive restrictions on alcohol advertising, sponsorship and promotion and **R**aising prices on alcohol through excise taxes and pricing policies [8]. Alcohol availability, prices, advertising, products and brands can be included in the larger concept of alcohol marketing. It is defined as a management process, from concept to customers, and it includes the four elements called the 4Ps of marketing: (1) identification, selection and development of a **P**roduct (i.e. taste, flavor, volume of alcohol, packaging, brand), (2) determination of its **P**rice (and discounts), (3) selection of a distribution channel to reach the customer’s **P**lace and (4) development and implementation of a **P**romotional strategy (i.e. advertising, sponsorship, digital media).

The impact of alcohol marketing on a young population, identified as vulnerable [13], is well described in the literature. In this subgroup, exposure to alcohol marketing is associated with early initiation, and an increase in drinking intentions, consumption and binge drinking [14–17]. It also leads to a normalization of alcohol consumption and an underestimation of the risks linked to consumption [18].

Drinkers with an AUD are also a vulnerable group, according to Babor et coll [13]. They are vulnerable to health damage [19, 20]; the relative risk of severe liver disease is very high in adulthood in men consuming 3 standard units per day between 18 and 20 years of age [21], and 90% of the deaths attributable to alcohol involve people with a daily consumption of 5 standard units per day or more [1].

Drinkers with AUD are potentially vulnerable to alcohol marketing, but very little work has been done on the links between exposure to alcohol marketing and alcohol consumption among people with an AUD. In the literature, some studies focused on heavy users of alcohol, i.e. those consuming more than the prescribed limits. These subjects reacted strongly to alcohol cues, and increased alcohol consumption is associated with increased attentional biases towards alcohol cues, which may increase subjective alcohol craving [22, 23]. Compared to non-heavy alcohol users, young heavy users of alcohol perceived greater alcohol consumption in alcohol ads but they also perceived this consumption to be responsible unless it was excessive [24].

Experimental studies have also been conducted using functional magnetic resonance imaging on small groups of adolescents (*n* = 15) meeting DSM-IV AUD criteria [25], or students (*N* = 46) regularly consuming alcohol with moderate or heavy drinking [26], or on adult heavy alcohol users (*n* = 20). These studies exposed participants to images of alcohol advertising or films, and concluded that adolescents and students meeting AUD criteria, or with higher than average alcohol consumption, had greater brain responsiveness when confronted with alcohol-related stimuli [25, 26], and greater psychophysiological responsiveness [26]. De Sousa Fernandes Perna et al. showed that in adult heavy alcohol users, advertising of alcohol products elicits striatal activation in the brain reward circuit [27].

Only two studies have analysed the effect of marketing of alcohol products on drinkers with an AUD who were seeking treatment [28, 29], but they were both conducted under experimental conditions. In 1993, Sobell et al. exposed 96 drinkers seeking treatment to television programs that included alcohol advertising. They showed that the more severe was the AUD, the less confident the patients felt about their ability to control alcohol craving and the desire to drink after the viewing [28]. Witteman developed a mixed methodology, used in a population of 80 drinkers with an AUD who were seeking treatment, and combined an experimental exposure to alcohol promotional films, and a prospective follow-up, over a 5-week period, where drinkers self-reported alcohol marketing exposure. They showed a high psychophysiological responsiveness to alcohol cues and a greater craving after alcohol cue exposure, proportional to the severity of the AUD. The drinkers reported being exposed to five alcohol marketing cues per day [29].

There is thus very little research on the impact of alcohol marketing in a population of patients with an AUD, particularly those who are seeking treatment. In a public health perspective, the impact of alcohol marketing on this population needs to be studied. It is a target for marketing strategies, as persons with AUD account for a majority of alcohol sales. In France 10% of people aged 18–75 years consume 58% of the alcohol marketed [30, 31].

Previous studies have focused on young populations of heavy drinkers [22–24, 32]. Only two focused on drinkers with an AUD who were seeking treatment [28, 29]. They were all conducted on a small number of participants and, in the majority of cases, under experimental conditions [24–26, 28, 29]. In addition, they used neutral images of alcohol (glasses, bottles), far removed from the attractiveness and complexity of real-world alcohol advertising cues. The other limitation of this research is that it only analyses exposure to one alcohol marketing tool, advertising (mostly through posters or promotional films), which does not reflect the broad scope of marketing [24–26, 28, 29, 32]. Finally, none of these studies included variables concerning beverage preferences. Drinkers with an AUD seeking treatment do have beverage preferences but the literature on this subject is sparse [33]. Socio-economic profiles differ according the preference [34, 35], and damage and modes of consumption also differ [33, 35, 36].

Drawing on these findings and limitations, the present study was designed via collaboration among researchers specialising in addictive disorders, in social marketing and in primary care. It is funded by the French National Cancer Institute (INCA).

To our knowledge, it is the first study to explore how drinkers with an AUD who were seeking treatment react to alcohol marketing in general, including beverage preference variables. The specific objectives of this study were 1/ to identify the main criteria influencing alcohol purchases 2/ to identify which alcohol marketing tools drinkers perceive as the most influential on their consumption, 3/ to identify factors making the product attractive, 4 / to determine whether they feel sensitive to the most visible marketing tool: advertising, 5/ to identify brands, products and beverage preferences. We also hypothesized that sensitivity to alcohol marketing would differ according to their beverage preferences among drinkers with AUD seeking treatment.

## Method

This study is a single center cross-sectional descriptive study. It was a preliminary step, part of a larger study funded by the French National Institute for Cancer Control (INCA) on the impact of alcohol marketing on people with AUD. The study protocol has been published previously [37].

Patients were recruited in the addictology department of Brest University Hospital from March 2019 to June 2019. This 4 months recruitment period was decided for feasibility reasons: it corresponded to the period of availability of investigators who included the patients. Participation in this study was offered to every drinker with an AUD who had a consultation in the centre specialising in addictive disorders during the inclusion period. Participants were not randomised.

### Study sample

The following were included: drinkers aged 18+, presenting a moderate or severe AUD, identified by an AUDIT (Alcohol Use Disorders Test) score (> 9) and confirmed by the DSM 5 (Diagnostic and Statistical Manual of Mental Disorders) criteria (at least 4 criteria, from the DSM alcohol use disorder section, in the previous year), who were seeking treatment during the inclusion period and who gave their written consent. Non-inclusion criteria were underage drinkers, vulnerable adults, and people who did not understand French language (written or spoken).

The inclusion of participants took place in the centre specialising in addictive disorders at Brest University Hospital. The study was proposed to patients corresponding to the inclusion criteria and attending the ambulatory center specialized in addictive disorders of the University hospital of Brest during the inclusion period.

### Questionnaire

The scientific committee (psychiatrists, physicians specialising in addictive disorders, researchers specialising in social marketing and methodologists) constructed a questionnaire, to be self-administered by patients with AUD. This questionnaire was tested on a sample of drinkers to ensure it was understandable and feasible. It was then modified according to the comments and concerns of interviewees and interviewers.

The final questionnaire was in the form of multiple-choice questions and four open-ended questions. Variables collected in the questionnaire are presented in Table 1. The Questionnaire is available as supplementary material.

**Table 1:** Variables collected in the questionnaire

**Alcohol marketing perception**	Criteria influencing the purchase of alcohol Factors making the product attractive Perception of the influence of alcohol marketing on their consumption Preferred brand and beverage preference (at initiation of alcohol consumption, at the moment the AUD appeared and current) Perception of the influence of alcohol advertising: recall of an advertisement for alcohol seen in the last 6 months Perception of the beverages entailing the most and the least risk
**Personal and familial history**	**AUD familial history** and beverage preferences in their family **Personal alcohol consumption** Conditions of alcohol initiation History of alcohol use disorders (duration) Beverage preference at alcohol initiation, when it became a problem and currently. DSM 5 criteria in the previous 12 months AUD main damage
**Sociodemographic variables**	gender/ age / working status / living single or in a couple

Concerning the marketing variables, criteria influencing the purchase of alcohol (buy alcohol somewhere), factors making the product attractive and perceptions of the influence of alcohol marketing on their consumption were collected. They were also asked if they had preferred brands and beverages, and about their perception of the influence of alcohol advertising: recall of an advertisement for alcohol seen in the last 6 months. Collected data are presented in Table 1.

Socio-demographic data was also collected (gender, age, working status, income, living single or in a couple) and familial history regarding alcohol consumption (familial beverage preferences, AUD family history). Personal history on alcohol consumption and AUD were collected: age at alcohol initiation, age at AUD onset, beverage preferences at each age, criteria for AUD according to the DSM, and main damage from their AUD (medical, sociofamilial, criminal offense related).

#### Ethics statement

Participants were informed and their written consent was requested. The study was approved by the Ethics Committee for the Protection of Persons from Brest University Hospital (B2019CE.16).

#### Statistical analysis

All statistical analyses were performed using R (R Core Team, 2019). A descriptive analysis of the data with calculation of percentages, means, standard deviations and prevalence was undertaken. Univariate, then multivariate, logistic regressions were undertaken to determine which variables were associated with each current beverage preference. To identify a profile of drinkers with an AUD seeking treatment and their sensitivity to marketing, we performed a multivariate logistic regression using each current beverage preference as binary dependent variables. Model fit was assessed using the Hosmer-Lemeshow test.

## Results

### Descriptive analysis

Ninety-one patients were included in the study. The descriptive analysis of the sociodemographic variables and the personal and family history of AUD are presented in Table 2.

**Table 2:** Descriptive data of the sample: Sociodemographic data and familial and personal history with alcohol

Age M (Min-Max)	46.2 (19–71)
***Variables***	***% (N)***
**Male**	73.6 (67)
**Female**	26.3 (24)
**Living alone**	65.9 (60)
**Urban**	84.6 (77)
**Rural**	15.4 (14)
**Having children**	67.0 (61)
**Stable housing**	94.5 (86)
**Over school leaving age**	46(*N* = 51)
**Income (monthly)**	
No professional income	35.0 (32)
Income under 1200 euros	9.9 (9)
Income over 1200 euros	45 (41)
No answer	9.9 (9)
**History of regular alcohol consumption in the family**	78.6 (71)
**History of AUD in the family**	80.8 (73)
**Beverage preferences in the family**	
Strong Beers	0.0 (0)
Standard-strength beers	26.4 (24)
Wine	72.5 (66)
Spirits	34.0 (31)
**Personal beverage preferences at the initiation of alcohol consumption**	
Strong Beers	0.0 (0)
Standard-strength beers	56 (51)
Wine	20.9 (19)
Spirits	27.5 (25)
**Personal beverage preferences at the onset of the AUD**	
Strong Beers	18.7 (17)
Standard-strength beers	26.4 (24)
Wine	38.5 (35)
Spirits	38.5 (35)
**Current severity of AUD (DSM 5 criteria)**	
Severe AUD	88 (80)
Moderate AUD	12 (11)
**Duration of AUD**	
Under 9 years	31.9 (29)
From 10 to 19 years	29.6 (27)
Over 20 years	35.2 (32)
No answer	3.3 (3)
**AUD-related damage**	
**criminal offence-related**^a^	54.8 (59)
**medical** ^b^	75.8 (69)
**socio-economic**^c^	88 (80)

^a^ Types of criminal offence-related damage: 41.8% had been arrested for drink-driving, 45% (41) banned from driving, 16.5 (15) had been convicted for violent behaviour, and 23% had been convicted for unspecified reasons

^b^ Types of medical damage: 57.1% (52) described an alteration of their general state of health, 27.5% (25) neurological damage, 26.4% (24) described liver or digestive damage, and 18.7% (17) dermatological damage

^c^Types of socio-economic damage: 58.2% (53) described isolation, 35.1% (32) financial damage, 42.8% (39) professional damage, and 52.7% (48) damage to marital relationship

### Marketing variables and beverage preferences

Current beverage preferences concerned wine for 39.6% (*n* = 36), standard-strength beers for 29.6% (*n* = 27), spirits for 27.5% (*n* = 25) and strong beers (over 6%) for 16.5% (*n* = 15) of the sample.

#### Influence of alcohol marketing

72.5% of the participants reported that they were not influenced by alcohol marketing, 24.2% reported being influenced and 3.3% did not respond to the question. However, when they explained their main criteria when buying a specific type of alcohol, they cited the price (39.5%, *n* = 36), accessibility (25.2%, *n* = 23), the brand (24.2%, *n* = 22) and alcohol percent (18.6%, *n* = 17), which are marketing elements. None of the participants considered packaging as a criterion when buying alcohol.

Regarding the marketing of “Products”, they were influenced by the type, the alcohol by volume and brand. 42.6% of patients had a preferred brand of alcohol. The reasons cited for these brand preferences were: taste (21%, *n* = 19), attractive price (8%, *n* = 8) and alcohol percent (5%, *n* = 5), while 64% (*n* = 57) did not justify their preferences.

Concerning advertising, 76.9% were able to recall alcohol advertising heard or seen in the previous 6 months. Among the participants who could recall advertising, the media they selected and reported were poster advertisements (43%, *n* = 30), and within this category, bus-shelter advertising posters were mentioned by 43% (*n* = 30); magazines were cited by 17% (*n* = 12) and television by 9% (*n* = 6).

In addition, 61.5% (*N* = 56) of the patients reported that the level of risk differed according to the type of alcohol. Alcoholic beverages considered the least likely to put them at risk were standard-strength beer (20.8%, *N* = 19), cider (20.8%, N = 19) and wine (19.7%, *N* = 18). The most likely to put them at risk, according to participants, were spirits (49.4%, *N* = 45), strong beer (12.1%, *N* = 11) and wine (6.6%, *N* = 6).

#### Comparative analysis

A comparative analysis was conducted to determine whether, according to gender or age, differences in marketing variables appeared in the study. Results are presented in Table 3. Men reported less being influenced by packaging than women (OR = 0.19, *p* = 1%). In comparison to patients aged 31 to 50 years, patients aged 51 years or more reported less both identifying a brand as attractive (OR = 0.29, *p* = 5%) and identifying the brand as the most influential criterion in alcohol purchase (OR = 0.35, *p* = 3%).

**Table 3:** Multivariate logistic regressions. Comparative analyses for marketing variables according to gender and age

Factors making the product attractive						
	Gender (reference: women)		Age (reference: 31–50 years)			
	Men	p	18–30	p	51 +	p
Brands	0.71 [0.25; 1.91]	0.50	4.46 [0.68; 88.2]	0.18	0.68 [0.27; 1.69]	0.41
Price	1.71 [0.62; 4.7]	0.29	4.16 [0.65; 82]	0.20	1.04 [0.41; 2.71]	0.93
Packaging	0.19 [0.046; 0.68]	0.01*	2.3 [0.26; 15.8]	0.41	0.59 [0.13; 2.42]	0.47
Alcohol percent	0.90 [0.32; 2.44]	0.84	0.55 [0.12; 2.65]	0.45	0.62 [0.24; 1.56]	0.31
Accessibility	1.08 [0.25; 3.92]	0.91	0.19 [0.03; 1.22]	0.07	1.43 [0.39; 6]	0.60
**Criteria influencing the purchase of alcohol**						
	Men	p	18–30	p	51 +	p
Brands	0.94 [0.30; 3.35]	0.92	1.32 [0.24; 6.2]	0.73	0.29 [0.08; 0.93]	0.05*
Price	0.98 [0.37; 2.72]	0.97	1.65 [0.35; 7.82]	0.52	1.15 [0.46; 2.89]	0.77
Alcohol percent	1.08 [0.33; 4.26]	0.90	0.50 [0.03; 3.33]	0.54	0.76 [0.23; 2.32]	0.63
Accessibility	0.99 [0.33; 3.21]	0.98	No data		1.31 [0.49; 3.54]	0.59
**Influence of alcohol marketing**						
	Men	p	18–30	p	51 +	p
**Influence of alcohol marketing**	3.68 [0.91; 24.9]	0.11	0.27 [0.01; 1.81]	0.25	0.35 [0.10; 1.07]	0.08
**Preferred brand**	2.52 [0.88; 8]	0.10	1.55 [0.33; 8.6]	0.59	0.35 [0.13; 0.90]	0.03*
**Recall of an advertisement for alcohol seenin the last 6 months**	1.09 [0.31; 3.44]	0.89	0.67 [0.13; 5.15]	0.66	0.73 [0.24; 2.28]	0.59

*p<0.05

#### Predictive variables associated with current beverage preferences

A multivariate logistic regression was performed using each current beverage preference as binary dependent variables to determine whether differences appeared as to the influence of alcohol marketing and for profiles according beverage preferences. Statistically significant and near-statistically significant predictive variables, and their associated odds ratios, are presented in Table 4.

**Table 4:** Multivariate logistic regressions. Predictive variables, statistically and near-statistically significant, associated with current beverage preferences among participants

***Beverage preference: beer***		
Criminal offence-related damage, conviction	**5.66 [1.58; 25.12]**	**0.013**
Alcohol by volume as the main criterion when buying	**4.14 [1.23; 16.15]**	**0.028**
Introduction to alcohol with wine	**0.16 [0.03; 0.74]**	**0.028**
***Beverage preference: spirits***		
Income > = 1200€	**4.81 [1.23.; 22.8]**	**0.032**
History of spirits consumption in the family	**5.18 [1.4; 22.9]**	**0.018**
Spirits consumption at AUD onset	**18.5 [5.05; 88.4]**	**< 0.001**
***Beverage preference: Wine***		
Type of media recalled: magazines	**8.48 [1.29; 87.9]**	0.041
Brand as the main criterion when buying	**0.15 [0.03; 0.55]**	0.007
Gender: male	**0.14 [0.02; 0.69]**	0.021
History of spirits consumption in the family	**0.19 [0.04; 0.75]**	0.026

People who mainly consumed beer (either standard or strong) were significantly more likely to report alcohol by volume as the main criteria when buying alcohol, and to report criminal offence-related damage. In contrast, they were significantly less likely to have started alcohol consumption with wine. The Hosmer-Lemeshow test did not detect any significant model misfit (*p* = 0.506, chi2 = 7.29, df = 8).

People who mainly consumed spirits were significantly more likely to have a net monthly income above 1200 euros, to have a history of spirits consumption in their family and to have been consuming spirits at AUD onset. The Hosmer-Lemeshow test did not detect any significant model misfit (*p* = 0.467, chi2 = 6.64, df = 7).

People who mainly consumed wine were significantly more likely to recall advertising in magazines and to be female. They were however significantly less likely to report the brand as the main criteria when buying alcohol, or to have a history of spirits consumption in their family. The Hosmer-Lemeshow test did not detect any significant model misfit (*p* = 0.14, chi2 = 12.25, df = 8).

## Discussion

### Exposure is underestimated by people with an AUD

Very few studies have focused on alcohol marketing exposure among patients with AUD seeking treatment, and none included beverage preferences.

72.5% of the patients with AUD reported that they were not influenced by alcohol marketing, but 76% of the patients recalled alcohol-related advertising in the previous 6 months. No significant statistical differences appeared according to gender or age for this variable. Alcohol marketing cues are unconsciously absorbed. The unconscious mental processes in consumers’ choices have been modelled by several authors [38–40]. According to these models, human behaviours are guided by automatic processes, including behavioural mimicry, trait and stereotype activation, and nonconscious goal pursuit [39]. Martin and Morich defined habits as a specific type of automaticity, and stated that, where habits are concerned, behaviours occur outside goals and intentions and that these behaviours are completely controlled by contextual stimuli [39]. Regarding the process of AUD, and the brain mechanisms underlying the profound disruptions in decision-making abilities in addictive disorders [41], it can be supposed that people with AUD behaviours are guided by habits which are, therefore, outside their conscious control.

### Types of media identified

Among the media mentioned, poster advertising, and particularly bus-shelter poster advertising, were cited by 43% of the sample. These poster advertisements are present in public places with pedestrian and road traffic, close to schools and therefore the risk of exposure is high. Magazines were also cited by 17% of the sample and, surprisingly, television by 9%. The Evin Law introduced into the French Public Health Code an exclusive list of media authorised for direct and indirect advertising of alcohol. Since then, alcohol advertisements have been banned on television screens [18, 42]. This unexpected result is consistent with a French study on adolescents, where 30.2% reported having been exposed to television advertising at least once a week [43]. One hypothesis is that they were referring to exposure to alcohol cues in films or TV series. In the literature, links between exposure to alcohol consumption in films in populations of people with an AUD have not been studied. In younger populations, it has been shown that it may be the cause of alcohol initiation among young people and its sustainability over time [44], and the appearance of alcohol consumption on the screen seemed to influence the desire to drink among young watchers, both positively and negatively, depending on how it was staged [45].

### Marketing factors making the product attractive and influencing alcohol purchases

Regarding the marketing of the “product” and the criteria that made the product attractive, the most frequently cited aspects were the type of beverage, the alcohol percent and the brand. In this study, 42.6% had a preferred brand, and those reporting a preferred brand were more often aged 19–50 years (*p* = 0.03). One specificity associated with women was identified: they reported significantly more often (58.4%) than men (41.6%) that packaging made the product attractive but it did not appear as a factor influencing alcohol purchases.

When buying alcohol, the main criteria involved were the price (39.5%), accessibility (25%), the brand (24%) and the alcohol percent (18%). No difference linked to gender or age was statistically significant for the criteria influencing alcohol purchases. In the literature, access to the product and price are two factors involved in the purchase of alcohol [46]. With regard to availability, a study in Finland showed that the presence of a large number of wine retail outlets near home increased wine consumption among men and women [47]. The effects of prices on sales are also well established [48]. Increases in prices tend to decrease problem drinking; the fact that problem drinkers spend more of their income on alcohol means they are more affected by price increases [49, 50]. Moreover, heavier consumers may respond to price increases by shifting their consumption location, where they are at risk, to a safer location such as home [48]. A group of French experts recently recommended an increase in alcohol price per gram to reduce alcohol damage [51]. A meta-analysis showed a significant effect of raising alcohol prices on the incidence of drinking; increasing the price of alcohol, and the taxes applied, is the main effective preventive measure for reducing alcohol consumption [52]. It has been estimated that adjusting the tax on beer to the rate of inflation from 1951 onwards would have reduced the number of deaths among young people aged 18 to 20 years by 15% [53].

### Beverage preferences and correlated variables

Spirits are the most extensively consumed forms of alcohol and they are defined as luxury products [54]. In our study, 27.5% reported having first started drinking with this type of alcohol, and 38.5% were consuming spirits at AUD onset; 27.5% currently consume more spirits than other forms of alcohol. Those who identified spirits as their preferred beverage were significantly more likely to have a higher income than wine or beer consumers, and also to have an history of spirits consumption in their family and to have started their alcohol consumption with spirits. In contrast, those reporting wine as their preferred beverage were significantly less likely to report a family history of spirits consumption. Familial transmission and heritability of AUD is well-known, even if the mechanisms still remain unclear [55]. Our results concerning the association between familial beverage preferences for spirits, the beverages consumed at initiation and the type of current beverage preference could raise the issue of the early learning and family transmission of beverage preferences, among patients with an AUD.

Wine was the most commonly consumed alcohol, after beer, by 39.6% of patients with AUD, it was also one of the most frequently consumed beverages at the onset of AUD for 38.4% of the patients, equal to spirits. These results contrast with marketing arguments which often use contrasting images: drinkers with AUD are caricatured as consuming spirits or strong beers while non-problem drinkers are portrayed as consuming wine. The significant variables associated with wine consumption were gender (more frequently women), and the recall by drinkers of alcohol advertising in magazines (OR = 53.21). Magazines are the main medium used for wine-related articles and advertising. However, it was not statistically possible to quantify this effect, so this result needs to be confirmed on a larger sample. People who identified wine as their preferred beverage were less likely to report the brand as the main criterion when they purchased alcohol. These results coincide with the wine industry’s marketing strategies in France, which focus on appellations and vintages, rather than brands.

Standard-strength beer had a considerable place in the initiation of alcohol consumption: 56% reported having first started drinking with standard-strength beer, while no patient mentioned strong beer. The proportion of standard beer consumed was lower at AUD onset, with a larger proportion of strong beer consumption taking over for 18.5% of patients. One of the hypotheses could be that there are changes in the criteria influencing the choice of alcohol bought in the course of the history of the disorder. Alcohol by volume was identified as the main criterion for purchasing alcohol (OR = 3.88) among drinkers identifying beer as their current beverage preference. A significant correlation with the presence of negative legal consequences was also identified. Certain studies had already found differences in damage sustained according type of alcoholic beverages consumed [56, 57]. A relationship between the type of beverage consumed and the likelihood of injury has been identified, probably not because of any intrinsic quality of the beverage, but attributable to differences in age, gender and the amounts consumed. Beer consumers tend more towards binge drinking which puts them more at risk for accidents and legal consequences [56, 57]. Finally, in our study, respondents were less likely to report having initiated alcohol consumption with wine.

In relation to their beverage preferences, participants were asked about the perceived risks when consuming different type of beverage. The sample had experienced a great deal of damage from alcohol consumption, 88% having developed a severe AUD, resulting in socio-financial, medical or criminal offence-related damage for 88, 75.8 and 54.8% respectively. In addition, 80.8% had history of AUD in their family. However 61.5% reported differences in terms of the perceived risks across the different types of alcoholic beverages. The risks seemed to be identified according to the alcohol by volume: spirits and strong beer were perceived as entailing the highest risk and standard-strength beer and cider carried the lowest risk. What was interesting in our study is that wine had a specific place: it was considered to be one of the beverages involving a lower risk for 19.7%, although for 6.6% of the sample, it was cited among the beverages entailing the highest risk. These findings could reflect the alcohol industry’s marketing strategies where the risks from drinking alcohol are not promoted, while highlighting the potential health benefits of alcohol consumption is a frequently used strategy [58, 59]. Wine, in particular, is targeted using this strategy, especially in France, partly for economic and cultural reasons. 4.6 billion litres of wine were produced in France in 2018, which amounted to 17% of world production. The volume of wine consumed in France is the second highest in the world [60], which is in line with the place of wine in the patients’ family habits in this study: 72.5% of participants reported that wine was the most frequently consumed alcohol in their families. From ancient times, wine has been portrayed in close association with food, and for many years, moderate and regular consumption of wine has been associated with health benefits, for which there is no scientific basis [61]. This induces misinformation and controversial debates, such as in 2018, in France, when Albert Hirsch and other public health representatives added their names to a column in a leading newspaper to remind the public that health damage from alcohol did not depend on the type of alcohol, but on the amount of alcohol consumed [62].

### Strengths and limitations

The main strength of this study is that it is an innovative topic, with a potentially powerful impact on public health, but with a lack of supporting data in the literature. The sample is representative of drinkers with an AUD seeking treatment, and comparable, in terms of the sociodemographic variables, to a national study [63].

The main limitation for our findings is type I error inflation due to the important number of statistical tests performed. These preliminary results should be received with caution until they can be confirmed in an independent sample.

The first bias is a selection bias because it was a single center study. A large majority of the patients (35.2% of the sample), presented severe AUD, lasting more than 20 years. The average age was quite old (46.2 years) with a broad range (from 19 to 71 years), and the majority of the sample was composed of men (73.6%). The sample was not large and this has limited the statistical analysis because of the probable lack of power.

The questionnaires were completed independently by the patient, and questions concerning past events may have involved a memory bias.

This was a preliminary study on a limited sample, and these results are not generalizable. These results have to be confirmed in a larger study, that will use a mixed-methodology in a multicentric sample of patients with an AUD.

## Conclusion

This study showed that vulnerable patients with an AUD were widely exposed to alcohol marketing cues, without being aware of this exposure. Many media used in alcohol advertising were cited by patients (posters, magazines, television..), and associated factors influencing the purchase of alcohol, such as price and availability, were widely identified., as were beverage preferences. These preliminary results, which need to be confirmed in a future study, are in line with recent French expert recommendations on alcohol regulation. There is a lack of information available to the general population, and to alcohol consumers in particular, on the risks involved in the consumption of alcoholic beverages assumed to entail lower risk, as well as on the thresholds and the justifications for these assumptions. Restrictions on alcohol marketing are a key factor in alcohol control and in risk reduction in AUD, and this study highlights the importance of reconsidering this issue in France, at a time when the legislation is weakened, to help limit exposure to stimuli among the most vulnerable.

## Supplementary Information

**Additional file 1.** Supplementary materials- Questionnaire of the study

## Data Availability

No additional data available.

## Abbreviations

AUD: Alcohol use disorder
AUDIT: Alcohol Use Disorders Test
DSM: Diagnostic and Statistical Manual of Mental Disorders
WHO: World Health Organisation
