# Supplementary material for: Alcohol use disorders, beverage preferences and the influence of alcohol marketing: a preliminary study
Source: Subst Abuse Treat Prev Policy. 2020 Nov 30;15:90. doi: 10.1186/s13011-020-00329-8 (PMC7706018; doi:10.1186/s13011-020-00329-8)
Supplement: Supplementary file 1 — Additional file 1. Supplementary materials- Questionnaire of the study [file 13011_2020_329_MOESM1_ESM.docx]

Information à l’étude et à l’utilisation des données.

Madame, Monsieur, Le questionnaire ci-joint que l’on vient de vous remettre est un autoquestionnaire, que vous pouvez, si vous le souhaitez, remplir seul maintenant et remettre dans l’enveloppe et l’urne dédiée dans la salle d’attente.

Cela vous prendra 5 à 10 minutes maximum. Les données sont totalement anonymes.

Les responsables et coordonateurs de la recherche sont Mme Beauvais Céline (interne) et Dr GUILLOU Morgane du CHRU Brest. L’objectif de ce travail est de mieux connaitre les facteurs de risque de consommation d'alcool en fonction du type d'alcool consommé.

Si en répondant à ce questionnaire, vous avez des questions ou des difficultés, **nous vous encourageons à en parler avec les membres de l’équipe d’addictologie** qui vous reçoit.

Dans le cadre de cette recherche, un traitement informatique de ces données va être mis en œuvre pour permettre d’analyser les résultats dans le respect de la confidentialité et du secret médical. Un fichier informatique de données va être constitué et les données seront identifiées avec un numéro de centre de soins et un numéro d’inclusion dans l’étude. Les données recueillies, y compris les données relatives à vos habitudes de vie vous concernant seront transmis au responsable de la recherche.

Vous êtes libre d’accepter ou de refuser de participer. Si vous acceptez, vous êtes libre de changer d’avis à tout moment sans avoir à vous justifier et votre décision ne portera aucun préjudice à la qualité de votre prise en charge.

-Age:

-Sexe :

**Concernant votre MODE DE VIE (entourer les réponses)**

- Comment vivez vous :

seul / en couple

- Avez-vous des enfants :

oui / non

nombre :

- Vivez vous : en ville / à la campagne?

- Avez vous obtenu le bac : oui / non

avez vous fait des études au delà du bac, si oui combien d'année après le bac : bac + ...

- Quelle est l'origine de vos revenus ?

-pas de revenus

-Revenus d'emploi :OUI / NON

si oui vos revenus sont ils au-delà du smic (1200 euros) : oui/non

- Autres revenus : AAH , RSA , invalidité ..

-Est ce que votre logement est stable : oui / non

**Concernant la place de l'alcool dans votre famille :**

- Avez-vous souvenir dans votre famille que la consommation d'alcool était régulière ? oui/non

- Quel type d'alcool était **principalement** consommé dans votre famille?

vin (rouge/ blanc / rosé)

champagne

cidre / chouchen

bière standard

bières fortes

Alcools forts : whisky / rhum / vodka/ cognac /gin / tequila /ricard/pastis

premix

Autres :

- Selon vous, au sein de votre famille, y a t-il des antécédents de consommation d'alcool problématique ( addiction / dépendance) ? OUI /NON

**Maintenant , nous allons vous poser des questions vous concernant :**

-Dans quelles circonstances avez-vous consommé de l'alcool la première fois de votre vie ? (entourer la réponse)

famille

amis

seul

travail

autre :

- Quel type d'alcool avez vous consommé la première fois ? (entourer la réponse)

vin (rouge/ blanc / rosé)

champagne

cidre / chouchen

bière standard

bières fortes

Alcools forts : whisky / rhum / vodka/ cognac /gin / tequila /ricard/pastis

premix

Autres :

- Depuis combien d'années votre consommation vous pose t'elle des problèmes?

..... ans

- Quel type d'alcool **consommiez vous le plus** au moment où votre consommation d'alcool est devenue plus problématique ? (entourer la réponse)

vin (rouge/ blanc / rosé)

champagne

cidre / chouchen

bière standard

bières fortes

Alcools forts : whisky / rhum / vodka/ cognac /gin / tequila /ricard/pastis

premix

Autres :

- Dans l'année passée, quel était **l'alcool que vous consommiez le plus** ? (entourer la réponse)

vin (rouge/ blanc / rosé)

champagne

cidre / chouchen

bière standard

bières fortes

Alcools forts : whisky / rhum / vodka/ cognac /gin / tequila /ricard/pastis

premix

Autres :

-Dans l'année passée, dans quel contexte consommiez vous le plus souvent de l'alcool ? (Entourer la réponse)

- A l'extérieur (bars, cafés ...)

- Chez des amis / voisins

- Au domicile

-Autre :

-Quels étaient les effets principaux que vous recherchiez avec vos consommations d'alcool ? (au choix, 2 maximum à entourer )

-Anxiolytique (diminuer le stress / angoisses)

-pour dormir

- pour oublier

- se stimuler

- se sentir euphorique, plus joyeux

- se sentir moins triste

- se soulager ses douleurs

- avoir plus confiance en soi / se sentir fort

- pour le plaisir du goût

-se sociabiliser : être plus en liens avec les autres

- autres :

**Concernant votre consommation d'alcool, dans l'année passée , pouvez-vous nous dire :**

1. Aviez vous l’impression de consommer de l'alcool en quantité plus importante ou sur une durée plus longue que ce que vous aviez envisagé initialement ?

OUI / NON/ Ne sais pas

2. Aviez vous déjà eu envie d’arrêter de consommer l'alcool ou de le diminuer sans y parvenir ?

OUI / NON/ Ne sais pas

3. Aviez vous eu l’impression de passer beaucoup de votre temps à :

-obtenir de l'alcool (aller le chercher), le consommer

-ou récupérer des effets de l'alcool (vous endort, vous ralentit, vous gène dans le quotidien sur de périodes longues..par exemple) ?

OUI / NON/ Ne sait pas

4. Aviez vous eu parfois l’impression de ressentir une forte envie de prendre l'alcool (envie irrépressible , compulsive , parfois irrésistible ) ?

OUI / NON/ Ne sais pas

5. Aviez vous eu l’impression que votre consommation d'alcool a entrainé pour vous une incapacité à remplir des obligations importantes de votre vie que ce soit au niveau professionnel, de votre vie privée, de votre vie de famille (Manquements, absentéisme, conflits, … ) ?

OUI / NON/ Ne sais pas

6. Aviez-vous diminué, abandonné des activités sociales, de loisirs, récréatives du fait de votre consommation d'alcool ?

OUI / NON/ Ne sais pas

7. Continuez-vous à consommer de l'alcool malgré le fait que cela entrainait ou aggravait des problèmes relationnels, ou sociaux ou familiaux dans votre vie ?

OUI / NON/ Ne sais pas

8. Continuiez-vous à consommer de l'alcool malgré le fait que cela entrainait ou aggravait des problèmes psychologiques ou physiques persistants chez vous ?

OUI / NON/ Ne sais pas

9. Aviez vous déjà consommé l'alcool dans des situations ou cela a pu vous mettre physiquement en danger (exemple : conduite etc.) ?

OUI / NON/ Ne sais pas

10. Aviez-vous eu besoin d’augmenter les doses d'alcool :

-soit pour obtenir le même effet ?

-ou soit par impression que les effets de l'alcool diminuaient en gardant les mêmes doses?

OUI / NON/ Ne sais pas

11. Aviez-vous déjà ressenti des signes de sevrage en cas d’arrêt ou de réduction de l'alcool (troubles du sommeil, cauchemars, irritabilité, anxiété, tremblements, perte appétit … )?

OUI / NON/ Ne sais pas

- Enfin, quelles sont les **principales conséquences négatives que vous percevez** de vos consommations d'alcool ? (entourer les réponses )

- Problèmes médicaux :

problèmes hépatiques (foie)

digestifs

neurologiques

dermatologiques (peau)

moins bon état général (perte d'appétit et de poids) / chutes / traumatismes ..

-sociaux :

problèmes financiers

isolement

problèmes professionnels

problèmes de couple

- judiciaires :

problèmes liés à l'alcool au volant

retrait de permis

violences liées aux consommations d'alcool

condamnations

- Autres :

**Nous allons maintenant vous poser des questions sur votre perception de l'alcool en général :**

- Quelles images et perceptions pourraient , selon vous, être associées à l'alcool que vous avez l'habitude de boire ?

- plaisir

-convivialité

-détente

-image de virilité

-féminité

- indépendance

- perceptions négatives

- Autres :

- Avez-vous une marque préférée d'alcool :

OUI /NON

Si oui : laquelle ?

Pourquoi ?

- d'après vous, avez vous l'impression que les différents critères ci-dessous interviennent dans votre choix lorsque vous achetez de l'alcool :

- **PRIX** : OUI /NON / NE SAIS PAS

-**MARQUE** : OUI /NON / NE SAIS PAS

- **PACKAGING** ( apparence / couleur/ forme de la bouteille): OUI /NON / NE SAIS PAS

- **LE DEGRE D'ALCOOL** : OUI /NON / NE SAIS PAS

- **ACCESSIBILITE** (accès facile dans les commerces) OUI /NON /NE SAIS PAS

- Parmi ces différents critères ci dessus, **lequel interviendrait prioritairement** dans votre choix? (entourer la réponse)

PRIX / MARQUE/ PACKAGING/ DEGRE D'ALCOOL/ ACCESSIBILITE

- Selon vous, quel (s ) types d'alcool sont potentiellement les plus dangereux / à risque ? ( au choix, entourer )

- **il n'y a aucune différence** : tous autant à risque

- il y a des **différences entre les types d'alcool** :

Si oui lequel selon vous est le plus à risque : (entourer )

vin (rouge/ blanc / rosé)

champagne

cidre / chouchen

bière standard

bières fortes

Alcools forts : whisky / rhum / vodka/ cognac /gin / tequila /ricard/pastis

premix

Autres :

lequel selon vous est le moins à risque : (entourer)

vin (rouge/ blanc / rosé) champagne

cidre / chouchen

bière standard

bières fortes

Alcools forts : whisky / rhum / vodka/ cognac /gin / tequila / ricard/pastis

premix

Autres :

- Pensez-vous que le marketing de l'alcool (présence et présentations de l'alcool dans les publicités, packaging , présence dans les films / séries de l'alcool , sponsoring sportif etc.) influence votre consommation personnelle d'alcool ?

OUI /NON / Ne sait pas

si oui, de quelle manière ? (pouvez vous nous en dire plus ? )

-Dans les derniers 6 mois, vous souvenez vous avoir vu une publicité pour de l'alcool ?

OUI / NON

-Si OUI, de laquelle vous souvenez vous le mieux ? (type de pub :Publicités affichées , Publicités radio , publicités magazine ... et pour quel alcool ? )

Avez vous idée de pourquoi cette publicité vous a marqué (e ) ?

-attractive

-type de produits vendus

-image perçue

-autres:

ON VOUS REMERCIE BEAUCOUP POUR VOTRE PARTICIPATION A CE TRAVAIL

VOUS POUVEZ DEPOSER LE QUESTIONNAIRE DANS LA BOITE PREVUE DANS LE SERVICE
